# A scalable high-throughput targeted next-generation sequencing assay for comprehensive genomic profiling of solid tumors

**DOI:** 10.1371/journal.pone.0260089

**Published:** 2021-12-02

**Authors:** Jeffrey M. Conroy, Sarabjot Pabla, Sean T. Glenn, R. J. Seager, Erik Van Roey, Shuang Gao, Blake Burgher, Jonathan Andreas, Vincent Giamo, Melissa Mallon, Yong Hee Lee, Paul DePietro, Mary Nesline, Yirong Wang, Felicia L. Lenzo, Roger Klein, Shengle Zhang

**Affiliations:** 1 Research and Development, OmniSeq Inc., Buffalo, New York, United States of America; 2 Research Support Services, Roswell Park Comprehensive Cancer Center, Buffalo, New York, United States of America; 3 Bioinformatics, OmniSeq Inc., Buffalo, New York, United States of America; 4 Laboratory Operations, OmniSeq Inc., Buffalo, New York, United States of America; 5 HemePath Molecular, Roswell Park Comprehensive Cancer Center, Buffalo, New York, United States of America; 6 Clinical Evidence Development, OmniSeq Inc., Buffalo, New York, United States of America; 7 Information Technology, OmniSeq Inc., Buffalo, New York, United States of America; 8 Medical Affairs, OmniSeq Inc., Buffalo, New York, United States of America; The Jackson Laboratory for Genomic Medicine, UNITED STATES

## Abstract

Timely and accurate identification of molecular alterations in solid tumors is essential for proper management of patients with advanced cancers. This has created a need for rapid, scalable comprehensive genomic profiling (CGP) systems that detect an increasing number of therapeutically-relevant variant types and molecular signatures. In this study, we assessed the analytical performance of the TruSight Oncology 500 High-Throughput assay for detection of somatic alterations from formalin-fixed paraffin-embedded tissue specimens. In parallel, we developed supporting software and automated sample preparation systems designed to process up to 70 clinical samples in a single NovaSeq 6000^TM^ sequencing run with a turnaround time of <7 days from specimen receipt to report. The results demonstrate that the scalable assay accurately and reproducibly detects small variants, copy number alterations, microsatellite instability (MSI) and tumor mutational burden (TMB) from 40ng DNA, and multiple gene fusions, including known and unknown partners and splice variants from 20ng RNA. 717 tumor samples and reference materials with previously known alterations in 96 cancer-related genes were sequenced to evaluate assay performance. All variant classes were reliably detected at consistent and reportable variant allele percentages with >99% overall accuracy and precision. Our results demonstrate that the high-throughput CGP assay is a reliable method for accurate detection of molecular alterations in support of precision therapeutics in oncology. The supporting systems and scalable workflow allow for efficient interpretation and prompt reporting of hundreds of patient cancer genomes per week with excellent analytical performance.

## Introduction

Next-generation sequencing (NGS) technologies for comprehensive genomic profiling (CGP) of solid tumor samples have been established as standard of care in the clinical setting for the management of patients with advanced cancers [1–5]. NGS provides the benefit of simultaneous evaluation of multiple variant types in multiple samples across multiple genomic locations in a single run making NGS the method of choice for efficient molecular analyses. Identification of molecular alterations and tumor properties by CGP can be used to guide targeted therapy, immunotherapy (IO), potential drug combinations, and suggest clinical trial options and off-label uses for existing FDA-approved drugs [6–10]. CGP by NGS also serves as a tumor-agnostic method to detect similar molecular alterations across histologies [11–14]. For biomarker discovery, CGP enables detection of novel molecular alterations that affect key biological pathway(s) and aid development of more effective therapeutics [15–18].

CGP by NGS can be performed using DNA and/or RNA for detecting different types of molecular changes including small variants (substitutions, insertions, deletions and indels), copy number alterations (gain and loss), and rearrangements (fusion and splice variant transcripts) [1, 3, 19]. Larger targeted panels that identify novel and emerging biomarkers have been developed to accommodate recent FDA approvals for TMB and MSI as genomic biomarkers of response to immune checkpoint inhibitors (ICI) in addition to the growing list of markers needed to select patients for novel targeted therapies [20–26].The initial connection between high TMB and response to ICI was achieved through whole exome sequencing (WES) studies of tumor and paired normal tissues. Recent studies show that TMB can also be calculated with targeted panels covering genomic content of at least 1 mega-basepair (Mb) with close correlation between the panel and WES at the clinically significant 0 to 40 mutations per Mb range [27–30]. Thus, targeted sequencing of 1–2 Mb provides a practical alternative for routine clinical use. Similarly, sequencing of genomic instability for MSI, and now homologous recombination deficiency (HRD), can be simultaneously accomplished with larger targeted panels using a tumor-only approach, thereby eliminating the need for matched normal tissue [31, 32].

Broader tumor-only gene panels that target hundreds of genes have the appropriate genomic content for such assessment, however, challenges still exist for high-volume CGP clinical testing, including protracted turnaround time for specimen acquisition, reliable sample, library, reagent and data management, limited sequencing throughput, and interpretation complexity [33]. Additionally, reliably assembling accurate, cohesive, evidence-based clinical reports that combine the alterations detected by simultaneous sequencing of DNA and RNA libraries from multiple variant callers, requires computational power as well as sophisticated bioinformatic pipelines and laboratory information management systems (LIMS) that include up to date knowledgebase content to support clinical interpretation [34–36]. To overcome these challenges, a balanced approach which factors into consideration the purposes for which the assay will be employed, including whether the panel is designed for therapeutic, diagnostic and/or prognostic testing, and the intended patient populations (i.e., advanced stage cancer patients or germline hereditary testing) is required to implement a CGP assay which efficiently and robustly generates clinically useful information. Given these challenges, there is a pressing need for clinicians and medical centers to partner with centralized molecular diagnostic laboratories for efficient and effective delivery of CGP services [37, 38].

In this study, we present our initial results of the Illumina TruSight Oncology 500 (TSO500^TM^) High-Throughput assay as a scalable CGP method to reliably detect and deliver clinically useful biomarker information in support of precision therapeutics in oncology. This assay provides broad genomic coverage by analyzing 523 cancer-relevant genes from both DNA and RNA in one integrated workflow from routine FFPE samples [39]. Using only 40ng DNA, this 1.97Mb NGS panel screens full coding regions of 523 cancer-related genes for small variants, 59 genes for CNA, TMB and MSI. Also, using only 20ng RNA, 55 select genes for fusions including *ALK*, *RET*, *ROS1*, *NTRK1*, *NTRK2*, *NTRK3*, *ERG*, *FGFR1*, *FGFR2*, *and FGFR3* and two splice variants (*MET* exon 14 and *EGFR* exon 2–7 skipping) are targeted for structural variant identification. We benchmarked and validated the assay against orthogonal validated NGS assays using the high-throughput NovaSeq 6000^TM^ sequencer by multiplexing up to 70 paired sample libraries. The study established analytical sensitivity, reproducibility, and limit of detection for clinically relevant mutations on a large set of FFPE tissue specimens across different tumor types and variable pathological characteristics.

The details regarding the analytical and clinical validation study and results of this panel for utility as a scalable test for routine comprehensive tumor profiling in a high-volume reference laboratory is presented. In addition, we describe the laboratory management system, standardized analytical pipeline and reporting systems established for sample processing, quality control, data analysis, and variant classification and interpretation. At the time of writing, the assay covered genomic profiling markers for more than 300 National Comprehensive Cancer Center (NCCN) guideline recommendations, all FDA-approved pan-cancer testing recommendations, and over 900 clinical trial selection markers across more than 30 solid tumor types (OmniSeq Knowledgebase® [accessed January 21, 2021]). The implementation of these advanced technologies from order to final report, enables robust, scalable comprehensive profiling of alterations in patients with advanced cancers to guide standard of care medical management and therapeutic selection. On the basis of the analytical validation studies, the TSO500 genomic profiling assay included in OmniSeq INSIGHT^SM^ has been approved by the New York (NY) State Clinical Laboratory Evaluation Program (CLEP) for use in our Clinical Laboratory Improvement Amendments (CLIA) certified, College of American Pathologists (CAP) accredited laboratory.

## Materials and methods

### Cell lines, contrived samples and tumor specimens

**Fig 1:**
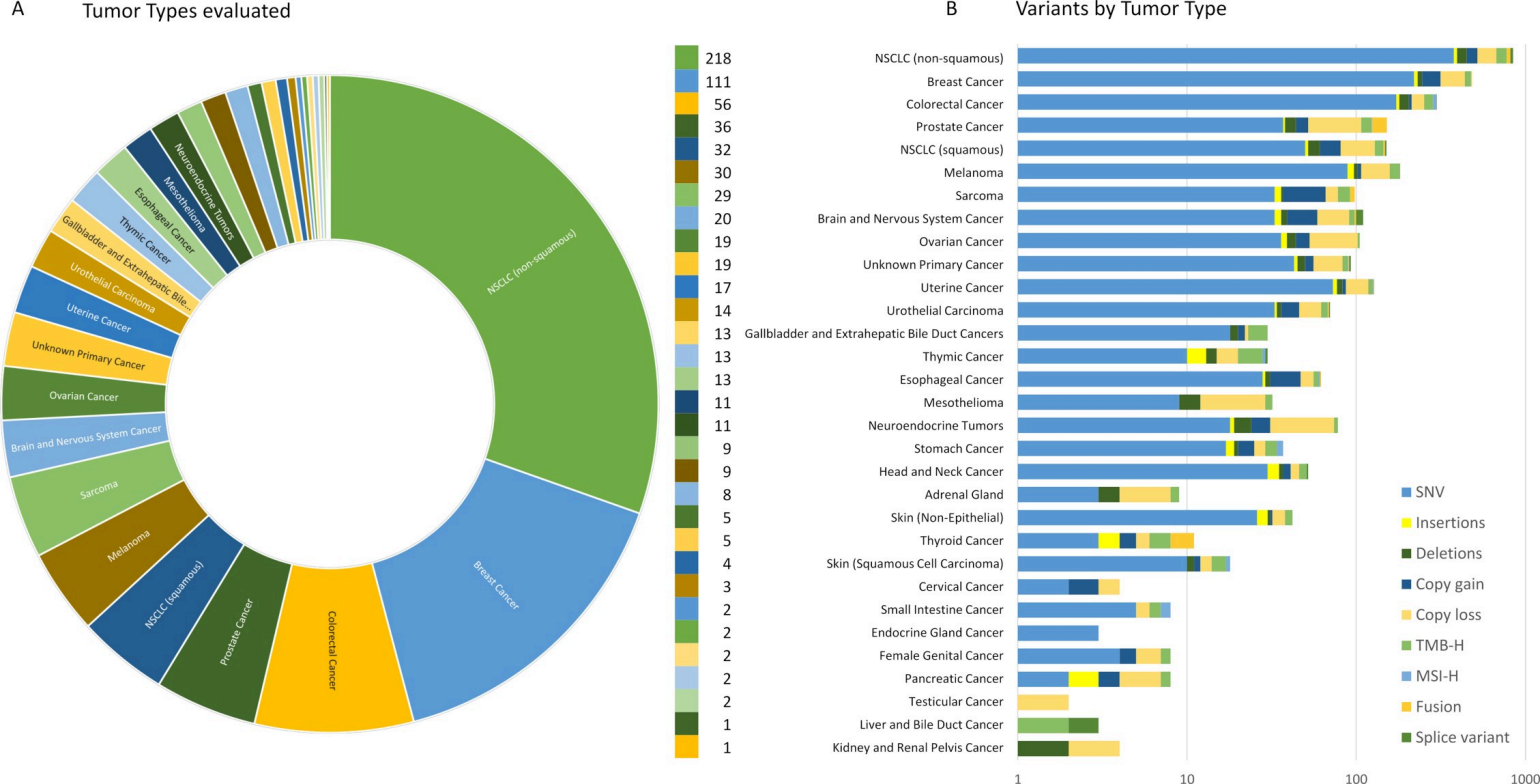
Validation cohort of 717 unique samples evaluated in TSO500 analytical validation study. A) Distribution of the 31 tumor types evaluated. Patients ranged in age from 5–99 years (406 female, 307 male, 4 unspecified). B) Summary of variant categories by tumor type.

To assess analytical performance of this assay, samples were selected from an inventory of remnant banked DNA and RNA isolated from formalin-fixed, paraffin-embedded (FFPE) clinical tumor specimens (406 female, 307 male, 4 unspecified) representing 31 tumor types (Fig 1, S1 Table in S1 File). Institutional Review Board approval was obtained from Roswell Park Comprehensive Cancer Center IRB prior to use of samples in the described validation studies. This study’s use of existing remnant de-identified clinical specimens was granted exemption as non-human subject research under protocol BDR #073116 and all data was anonymized prior to initiation of the described study. Specimens were procured from 2016–2020, and included DNA and RNA from tumor resections, needle core biopsies, and cell blocks from fine needle aspirations. The specimens were chosen to include a wide range of representative variant types for each class of alteration (substitutions, insertions and deletions, copy number alterations, and fusion/splice variants) in various genomic contexts across a broad selection of genes as well as analysis of genomic signatures including MSI and TMB (S2 Table in S1 File). These variants were originally identified by independent analytically validated NGS, PCR, FISH and Sanger sequencing assays. In addition, two commercially available contrived reference samples were used to assess analytical sensitivity, precision and assay processing as a run control. These include the Horizon Discovery Structural gDNA Multiplex Reference Standard (Cat# HD753) which is a cell line-derived control providing validated copy number amplifications, large insertions/deletions, and single base substitution variants within AMP/ASCO/CAP Tier I and II guideline positions and regions of specific GC-content (high vs. low), and the Seraseq® FFPE Tumor Fusion RNA v4 Reference Material (Cat# 0710–0496), which contains multiple engineered fusions and splice variants. To study assay specificity, DNA and RNA prepared from FFPE archived HapMap GM12878 (ATCC) and GM24385 (SeraCare Cat # 0710–1580) were used as wild type reference materials. To study analytical performance for the rare RNA fusions, four rearrangement positive cell lines were obtained from ATCC and used to assess RNA-seq performance: HCC-78 (ROS1), KM12 (NTRK1), LC-2/ad (RET), and SU-DHL-1 (ALK).

### Panel content and use

The TSO500 assay is an NGS-based in vitro diagnostic assay for the detection of genomic variants and signatures in FFPE tumor tissue (Illumina, Inc.). DNA is sequenced to detect small variants (single and multinucleotide substitutions, insertions, deletions and indels), including genes leading to homologous recombination repair defects (HRR/HRD), copy number alterations (gains and losses), as well as analysis of MSI and TMB. RNA is sequenced for detection of fusions and splice variants in select genes. The resultant information is intended for use by qualified health care professionals in accordance with professional guidelines in oncology for management of patients with solid neoplasms, and is not conclusive or prescriptive for labeled use of any specific therapeutic product.

The TSO500 assay is an enrichment-based targeted NGS assay comprised of reagents to generate libraries from DNA and/or RNA isolated from FFPE tissue of solid tumors encompassing multiple cancer types. The assay can be performed using DNA and RNA, DNA only, or RNA only. The assay employs library construction using unique molecular identifiers (UMIs) and hybridization-based capture of coding exons from 523 cancer-related genes, copy number alterations for 59 genes, select gene fusions with known and novel partners, and splice site variants from 55 genes (S3 Table in S1 File). The DNA-sequencing component targets >9,200 regions totaling >1.94M basepairs of DNA. This includes target regions specific for detection of copy number alteration (n = 59 genes), potential MSI unstable sites (n = 130), as well as specific identity indels and SNPs (S4 Table in S1 File) that serve as specimen identifiers and are evaluated as DNA contamination detection controls.

The RNA-sequencing component uses 1,319 regions averaging 270 bp for hybridization capture of 55 select genes, totaling 355,762 basepairs of transcribed sequence (S5 Table in S1 File). The RNA baits are designed to capture target genes and both known and novel fusion partners and splice variants.

### Assay workflow and systems

**Fig 2:**
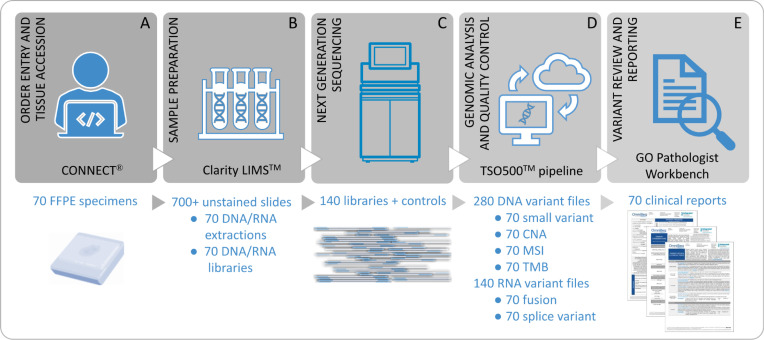
TSO500 workflow and supporting systems designed for a 70-sample run of routine FFPE tumor tissues. A) CONNECT® supports order entry, specimen collection, sample accessioning, and results transmission through use of a Web Portal. B) Clarity LIMSTM manages the test specific lab workflow including Sample Preparation from Tissue QC through library preparation. C) High-throughput next-generation sequencing of 140 matched DNA/RNA libraries on NovaSeq 6000TM Sequencing System. D) The TSO500TM pipeline analyzes the genomic data to generate qualified variants. E) Variant annotation, review, treatment assignment, pathologist signout and clinical reporting in the GenomOncology Pathologist Workbench.

The laboratory workflow utilizes several software systems to support the high-throughput CGP assay (Fig 2). First, the overall assay system was defined and optimization and feasibility testing was undertaken using a quality systems approach (ISO 13485:2016) and followed by document design control. The assay workflow includes order entry, specimen and preanalytic processing, nucleic acid extraction, NGS, data analysis, and generation of a clinical report. Supporting IT systems for order entry (CONNECT^®^), preanalytical and analytical workflows (Clarity LIMS^TM^), analytical pipeline (TSO500 pipeline), and variant review and reporting (GenomOncology Pathologist Workbench) were defined and documented as part of the design history file. Alterations are classified for clinical significance in the context of the patient’s specific tumor histology following joint consensus recommendation from the Association for Molecular Pathology, American Society of Clinical Oncology, and College of American Pathologists (AMP/ASCO/CAP) guidelines based on clinical evidence in the OmniSeq knowledgebase at the time of reporting [40].

### Assay validation overview and statistics

The following performance characteristics were established for the TSO500 assay: accuracy (concordance), precision, and limit of detection (LoD) at the variant or biomarker level, across all mutational types (small variants, CNAs, Fusions, Splice variants, MSI, and TMB). The analytical validation studies were performed followed NY State CLEP Oncology–Molecular and Cellular Tumor Markers: Next Generation Sequencing (NGS) guidelines for somatic genetic variant detection [41], FDA guidance documents, the CAP Molecular Pathology Checklist, and the AMP and CAP joint consensus recommendation [42]. Specimens tested in the study may have more than one mutation present in the tumor.

The data analysis for concordance is based on the binary classification result reported by the test and the reference assay as described [43]. The TSO500 assay results are qualitative which consists of positive (reported) or negative (not reported) results. The test is designed to determine whether a variant is present or absent, at the determined thresholds of detection. The numerical underpinning (variant allele frequency (VAF), fold change (FC), TMB Value, and fusion reads) of each component were used to explore concordant and discordant results between the TSO500 assay and the comparator test results. Statistical measures of agreement (Positive Percent Agreement (PPA) and Negative Percent Agreement (NPA)) were defined following common 2 x 2 table format for reporting results comparing a new test to a non-reference standard [44]. Pearson correlation (R) was used to measure the linear correlation of TMB and copy gain FC values, and coefficient of determination (R^2^) was calculated to estimate the strength of the relationships between the underlying TSO500 assay and comparator assay values for copy gain FC and TMB.

Reproducibility was assessed by calculating closeness of agreement for representative assay-reportable variants obtained under changed conditions, including within (intra-) and between (inter-) runs, operators, days, instruments, reagent lots and barcodes. Precision studies were performed for 131 specimens from common aliquots of extracted nucleic acid, with 8–53 replicates each depending on study and sample availability. Together these samples represented a range of SNVs, indels, copy gains, copy loss, fusions, splice variants, MSI, and TMB. The samples were tested by 3 different operators across 13 sequencing runs using 2 sequencing instruments, in addition to rotation of library barcodes and reagent kit lots. Panel-wide reproducibility across these variables, determined by positive and negative call rates, was assessed for each positive variant detected across all replicates. The positive and negative call rates determine the degree to which the repeated sequence analysis gives the same result across qualified genomic positions in the panel stratified by variant class and various allele frequency thresholds. To analyze the imprecision caused by changed conditions, the average positive agreement (APA) and average negative agreement (ANA) rates were assessed across all variables. The data are stratified by variant type and evaluated for overall, within-run, between days, between operators, between instruments, and between reagent lot concordance. The APA was calculated as the average positive call rate for a variant type (SNVs, insertions, deletions, copy gain, copy loss, MSI, fusion and splice variant) across all replicates of a given condition. The ANA is calculated as the average negative call rate for a variant type across all replicates of a given condition. Due to the quantitative result of mutations per megabase, TMB precision was assessed using % coefficient of variation (CV) of the TMB score across test sample replicates for samples considered as consensus TMB-High, using the ≥10 Muts/Mb threshold [45].

For SNVs and indels, the limit of detection (LoD) of the assay is defined as the variant allele fraction at which 95% of replicates across all replicates for a variant class are reliably detected. For TMB, MSI, CNA, fusions and splice variants, the LoD is defined as the minimum estimated percent tumor purity required to achieve a ≥ 95% call rate. All LoD studies are performed with the minimum recommended input of 40ng DNA and 20ng RNA. To estimate the LoD and associated 95% exact confidence interval (Clopper-Pearson) across the variant classes, the hit rate approach was utilized as per Canchola and Hemyari [46]. The hit rate approach examines the positive hit count at the dilution levels, generating a percent detection table along with associated 95% exact confidence intervals. LoD from the hit rate approach is defined as the lowest level with 100% hit rate (worst scenario).

### Tissue QC/preanalytical considerations

5μm sections were cut from tumor and cell line FFPE blocks and qualified by three board-certified anatomical pathologists for tissue quality following hematoxylin and eosin staining and microscopic review. Tissue regions with an estimated >20% tumor purity and estimated <50% necrosis were collected for nucleic acid extraction. Regions identified by the pathologist were used as guides to scrape tissue. In general, 5–20 unstained slide sections cut at 5μm, with and without tumor microdissection, are required to achieve the minimal assay requirements for DNA (40ng) and RNA (20ng) input. Genomic DNA and total RNA were simultaneously extracted using the truXTRAC FFPE extraction kit and LE220-plus Focused-ultrasonicator (Covaris, Inc., Woburn, MA), as previously described [47], integrated with the Lynx robotic liquid handler (Dynamic Devices, Phoenix, AZ) for automated reagent transfer, purification and elution. Extracted RNA and DNA were quantified using the Qubit dsDNA HS Assay Kit for DNA and the Qubit RNA HS Assay Kit for RNA. Genomic DNA was sheared using the LE220-plus instrument to 100–150 bp fragments prior to library preparation.

### Capture protocol and library preparation for NGS sequencing

Libraries were prepared using the minimum input requirements of 40ng DNA and 20ng RNA, unless otherwise indicated, with TSO500 assay (Illumina, Inc.) reagents following the manufacturer’s instructions [48]. In brief, RNA was reverse transcribed to cDNA using TruSight Oncology HT RNA Library Prep Kit (Illumina). Libraries were then prepared for each sample using a probe pool (one for DNA and one for RNA) to generate a uniquely barcoded library with UMIs. Following hybridization-capture, bead clean-up and library normalization, 36–72 matched DNA and RNA libraries were combined in the final library pool containing 80% DNA and 20% RNA, for a total of up to 144 barcodes. Paired-end sequencing was performed using the NovaSeq 6000 on the S2 flow cell. Studies were performed using up to 100ng DNA and RNA input to demonstrate reproducibility of results using variable input amounts typical of routine clinical testing. A water no template control (NTC) and the RNA and DNA contrived reference samples were included in each run as assay run controls. All wet laboratory procedures and NGS for the validation studies were performed by three trained operators following SOPs based on the manufacturer’s instructions (Illumina). The laboratory workflow, processes and samples were managed and tracked using the Clarity LIMS (Illumina; v5.1.5.3).

### Bioinformatics and data analysis pipeline

#### Demultiplexing, read alignment, UMI collapse, variant calling

Primary analysis including image processing, base calling, and quality scoring was performed on the NovaSeq6000 system using onboard Real Time Analysis (RTA) software (Illumina). Secondary analysis for run-level, sample-level and variant-level quality metrics was managed using Airflow (Apache; v1.10.0) and the TSO500 pipeline (Illumina; v2.1.0.60) for validation of run processing and quality control, demultiplexing, FASTQ processing, alignment, and DNA/RNA variant calling. Genome alignment was performed using the hg19 reference genome.

For DNA samples, error correction, small variant calling, copy number calling, TMB calculation, MSI status determination, and quality control was performed using the TSO500 DNA bioinformatics pipeline [39]. The assay utilized UMI to identify singular targets enriched for sequencing [49]. Simultaneous UMI read collapsing and error correction procedures were used on the raw sequencing reads for removal of duplicate reads, sequencing errors, and FFPE deamination artifacts. TMB was calculated based on eligible synonymous and non-synonymous variants divided by the size of the panel successfully sequenced (maximum 1.33 Mb exonic sequence), and reported as mutations per megabase (mut/Mb). CNA analysis for select gene gain (amplification) and loss (deletion) calling was performed using the CRAFT copy number variant caller (Illumina). CNA performance requires sufficient target regions on genes of interest, therefore the assay is limited to 59 genes for CNA analysis. MSI status was determined using Hubble (Illumina) by analyzing 130 targeted homopolymer sites for evidence of instability relative to a baseline derived from an independent cohort of normal samples. The proportion of unstable MSI sites to total evaluable MSI sites is reported as a sample-level microsatellite score.

For RNA samples, fusions and splice variants were identified using GENCODEv19 within the TSO500 pipeline. The Manta fusion caller was used to detect, assemble, and score fusions for 55 select genes. Splice variant calling is performed for the genes *EGFR* and *MET* using an algorithm to identify reads in these genes that span candidate splice junctions identified during alignment. Splice variants are compared against a database of known transcripts and a splice variant baseline of splice variants found in normal (non-neoplastic) FFPE samples from different tissue types.

#### Filtering based on technical characteristics

The small variant workflow was able to detect SNVs and indels below 2% VAF. However, based on a feasibility study a threshold of 2% VAF for Tier I SNVs and indels was established. For all other SNVs and indels, (i.e., Tier II and Tier III), a 5% VAF threshold was established for detection. Positive calls for small variants also required a minimum of 4 unique variant reads. Indel detection was restricted to <25bp of sequence alteration. Reportable Tier IA SNVs and indels were required to satisfy quality metrics that evaluated coverage and data completeness. Select Tier I SNV/indels were reported as indeterminate when 1) no evidence of the alteration was found, but minimum coverage was not met to support the verified limit of detection, or 2) reads suggesting the possible presence of the alteration were observed, but minimum coverage thresholds were not met to report the variant.

TMB was determined using the small variant output of 520 genes (excluding HLA-A, HLA-B, and HLA-C) and dynamically adjusted per sample based on sequencing depth using non-germline synonymous and nonsynonymous variants with ≥5% VAF.

Hubble assesses 130 potential sites for MSI. However, the total number of sites that can be successfully evaluated varies between samples. In order to be assessed, a homopolymer region was required to have a minimum of 60 reads spanning the site. To report MSI results, samples were required to have a minimum of 40 qualified sites. The proportion of unstable MSI sites to total evaluable MSI sites in a specimen is calculated as a microsatellite score. This score was evaluated against a pre-defined threshold to determine whether the sample was reported as MSI-High (>20% MSI unstable sites) or MS-Stable (≤20% MSI unstable sites).

Each CNA was reported at the gene level in the form of fold change (FC) on normalized read depth in a testing sample relative to the normalized read depth in diploid genomes. For copy gain, a FC ≥3.2 is considered as copy “gain” and FC = 2.2-<3.2 as copy “gain–indeterminate”. A 2.2x FC is equivalent to 10 copies in a tumor at 30% estimated tumor purity. Gene copy number gains for *CCND1*, *CCNE1*, *CDK4*, *CDK6*, *EGFR*, *ERBB2*, *FGFR1*, *FGFR2*, *KIT*, *KRAS*, *MET*, *MDM2*, *MYC* and *PIK3CA*, and gene copy number loss for *ATM*, *BRCA1*, *BRCA2*, and *PTEN* were validated in this study. A FC ≤0.5 was considered to be Copy “loss” and FC >0.5–0.7 Copy “loss-indeterminate”. A 0.5x FC is equivalent to 0 copies (somatic homozygous deletion) in a tumor at 50% tumor purity. Copy “gain-indeterminate” was reported as “Testing suggests the possibility of an increased copy number of gene X. However, results did not satisfy the reporting criteria for the assay. Additional testing using an alternative method is recommended if clinically indicated.” Copy “loss-indeterminate” was reported as “Testing suggests the possibility of an increased copy number of gene X. However, results did not satisfy the reporting criteria for the assay. Additional testing using an alternative method is recommended if clinically indicated.”

Candidate fusions were annotated as GeneA-GeneB when ≥5 unique candidate gene fusion reads were detected. Fusions for *ALK*, *FGFR3*, *NTRK1*, *NTRK3*, *RET*, and *ROS1* were validated in this study. Splice variant calling for *MET* Exon 14 and *EGFR* Exons 2–7 skipping required ≥8 unique reads at specific candidate exon splice junctions.

#### Variant annotation, clinical significance classification and reporting

Variant files were exported from the TSO500 pipeline and annotated using the Pathologist Workbench (GenomOncology; release 6289, rules 0009O, annotation pipeline 17). Intergenic, intronic, non-coding, and synonymous variants are removed with the exception of splice site mutations (+/- 1/2 in the intron). Post-bioinformatic classification of variants for final results reporting happens in two determination stages: 1) reportability, which is concerned with the quality and somatic nature of a genomic alteration, and; 2) matchability, which is concerned with the pathogenicity of the variant and the clinical efficacy of any therapeutic response association, either alone or as part of a combined profile of 2 or more reportable markers. Pre-defined rules are executed in the Pathology Workbench to first determine variant reportability, then variant matchability, based on the OmniSeq Knowledgebase®, for both positive and negative/wild type variant results (S6 Table in S1 File). The OmniSeq Knowledgebase includes therapeutic associations (sensitivity and/or resistance/lack of benefit) for all TSO500 tested variant categories included in FDA approved drug label indications, professional practice guidelines, and as selection criteria for clinical trials. A molecular pathologist signs out the final report, in which marker/drug/tumor type/response therapy associations for matchable variants are provided in the context of the patient’s primary tumor type in 5 groups—clinical benefit in the tumor type tested, resistance/decreased response in the tumor type tested, clinical benefit in other tumor types, or clinical trials. AMP/ASCO/CAP tier strength provided (IA, IB, IIC, or IID, with tier IA being the highest and covering FDA drug labels/professional guidelines) to clearly show the clinical significance of each therapy association. Matchable variants with no identified therapy associations for a specific tumor type at the time of the report are listed as such, while reportable, non-matchable variants are listed as variants of unknown significance (VUS) in the report appendix.

#### Quality metrics: Run, assay, sample

To ensure quality results, an ISO 13485 quality system and assay specific quality control (QC) metrics were developed in the OmniSeq laboratory. Thresholds for acceptability were set for run, assay, sample and analyte level quality review in the Pathologist Workbench (S7 Table in S1 File). Run level QC metrics evaluated the sequencing performance of two NovaSeq 6000 instruments by assessing the percentage of reads with a quality score equal ≥30, and total percentage of reads passing filter. Assay QC was evaluated by reviewing no template control (NTC) and Run Control RNA and DNA samples which were included and taken through the entire testing process (including sequencing) to verify that there was no contamination across samples and reagents (NTC), and to verify that low percentage variants were consistently identified. Sample QC was evaluated for several DNA and RNA specific criteria and were applied to all validation samples. SNV, indels and TMB shared common QC metrics and thresholds that were independent from the CNA and MSI variant class QC metrics. There are situations in which a DNA sample can fail one of the QC metrics and pass the others resulting in partial variant class reporting. Libraries generated from DNA samples were evaluated for potential contamination by DNA from other samples (foreign DNA) using a combination of a contamination score and a contamination p-value as provided by Illumina. In contaminated samples, some germline variants (identity indels and SNPs) have VAF that deviate from expected values of 0%, 50% or 100%. An algorithm computes a log likelihood score across all common and identity SNP positions where SNV calls were reported. The larger the contamination score, the more likely there is foreign DNA contamination. Samples failing to meet QC requirements were flagged for review for possible copy number aberrations or sample contamination with decision to either fail or accept DNA sequencing results (i.e., small variants, gene gains, TMB score and MSI status).

If a sample failed because of poor sequencing quality that affects all DNA or RNA variant class QC requirements, the respective DNA or RNA library preparation were repeated from library preparation. Individual failed DNA QC metrics for specific variant classes were not considered sample failures, but were instead flagged as analyte failures. A flagged sample proceeded to manual data review in the PWB at which point the alteration was accepted or rejected. If necessary, sample sequencing was repeated using re-normalized libraries. The study allowed a maximum of 1 round of repeat testing. The initial quality control thresholds were recommended from the vendor and established during the feasibility studies.

#### Statistical determination of coverage requirements

A power analysis to compute the sequence coverage or total number of unique UMI collapsed reads needed to detect a mutation with true underlying mutation frequency 2% or greater was performed. Statistical power was estimated based on a requirement of 4 UMI-supported mutant observations to make a positive call. Sequence coverage of >400x provides 95% statistical power for detection of true mutations at 2% VAF (95% CI, 0.8% - 3.5% VAF). For mutations with 5% underlying VAF, sequence UMI coverage of >150x provides 95% statistical power for detection (95% CI, 2.0%-8.6% VAF). To confirm exon coverage estimates, UMI collapsed sequence coverage was evaluated across a 250 FFPE sample cohort using 40ng DNA input and a 72-sample library pool batch size.

## Results

### Panel design and target region coverage

**Fig 3:**
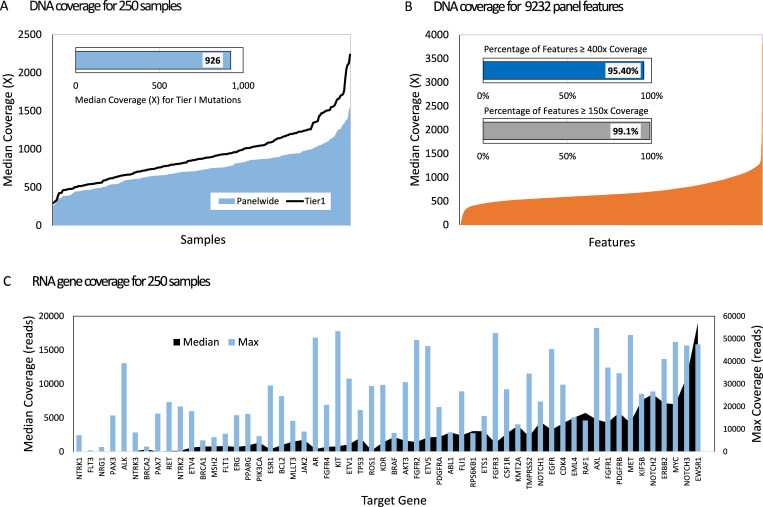
TSO500 summary sequencing metrics for 250 FFPE samples using standard 70 paired sample sequencing run and 40ng DNA and 20ng RNA inputs. A) DNA median coverage (UMI reads) across samples for all positions (blue) and Tier1 mutation positions with clinical significance (black line). B) DNA median coverage (UMI reads) across 9,232 TSO500 targeted genomic features. C) RNA mean and maximum coverage (blue bars) across 55 target genes.

DNA sequencing statistics were calculated for individual exons across a cohort of 250 samples from 27 tumor types to evaluate target coverage. The average median UMI collapsed coverage across all targeted regions for the FFPE samples was 750x (SD = 385), which is nearly 5x the coverage depth required for 95% statistical power for detection of 5% VAF. Regions with somatic Tier I mutations exhibited median UMI sequence coverage of 926x (median range 294 – 2237x), well above the 400x coverage depth required for 2% VAF calling as determined from our power calculation (Fig 3A). The DNA sequencing resulted in 95.4% of all panel features (8810 of 9232) sequenced to a median depth of 400x or greater (Fig 3B). Greater than 99% of targeted regions (9152 of 9232 total regions) were sequenced to a depth of 150x or greater (S8 Table in S1 File). 4,223 nucleotide positions in 72 genes consistently had depth below 80x and were blacklisted and removed from variant analysis and reporting. Greater than 80% of the blacklisted positions belong to 30 exons with nearly complete lack of coverage (S9 Table in S1 File). No clinically meaningful regions with current Tier I variants or somatic hotspot mutations were included in the blacklisted positions. The removal of the 4,223 low coverage nucleotide positions resulted in 1,975,834 total targeted bases.

Two reference materials, HapMap GM12878 (ATCC) and GM24385 (SeraCare) FFPE tissue with known backgrounds were evaluated to provide empirical evidence that analytical specificity was maintained using the established pipeline thresholds and filters. Specificity was observed at 100% with no CNAs, fusions, splice variants, MSI and TMB reported across qualified replicates (GM12878 = 24 and GM24385 = 7) for each standard. For Tier I variants, the rate of false positives was 0%. The false positive rate for panel-wide SNVs across the 1,975,834 basepair positions for GM12878 was 0.00047% (n = 225/47,420,016) and for GM24385 was 0.00043% (n = 59/13,830,838). For MSI-H (qualitative call), the false positive rate was 0 (n = 26). For TMB, the false positive rate (defined as TMB ≥10 Muts/Mb) was 0%. There were no false negative calls for MSI-H or TMB-High.

RNA sequence coverage was also evaluated at the gene level for each of the 55 target genes. The overall median depth of unique read RNA coverage for the 250 FFPE samples was 22,675 and ranged from 0–1818,978 (Fig 3C). Each gene was observed with expression positive read counts demonstrating probe efficiency to capture and detect all putative fusion targets. Even in the absence of an activating fusion, the expression levels of the target genes supports the >9,000,000 total RNA reads quality threshold that generates unique read sequencing depths adequate to confidently call low depth alternate fusions and splice variant reads in a background of baseline expression.

### Overall NGS data quality

Optimization and feasibility studies performed prior to the validation demonstrated that up to 72 DNA and RNA samples could be pooled per NovaSeq 6000 sequencing run using the S2 Flow cell, and achieve the depth of coverage required for >95% sample to achieve >400x coverage per specimen. During feasibility studies it was also determined that the minimum nucleic acid input required to consistently achieve key performance characteristics using FFPE samples, was 40ng of DNA for the DNA-Seq test mode and 20ng of RNA for the RNA-Seq test mode. Performance characteristics were established at these inputs using 583 DNA and 477 RNA derived from a wide range of FFPE tissue types, including resections, needle core biopsies, and cell blocks from fine needle aspirations. At the minimum nucleic acid inputs and maximum sample multiplexing, there was a 96.7% overall success rate for DNA and 91.4% for RNA (S10 Table in S1 File). There was no instance of a sequencing run QC failure. Additionally, NTC, RNA and DNA run controls met all assay level QC metrics (100%). In total, 4,112 libraries (2,119 DNA and 1,993 RNA) were sequenced with >95% success rate.

### Accuracy (concordance)

The analytical accuracy of TSO500 was evaluated using clinical FFPE samples obtained from patients with a variety of tumor types to assess all variant classes. Data was aggregated at the variant level for SNVs and indels, gene level for copy gain, copy loss, fusions and splice variants, and case level for MSI and TMB. The detection of alterations by TSO500 profiling was compared to results of orthogonal validated NGS, WES and RT-PCR assays and are collectively termed validated NGS assays (vNGS).

### Small variants

**Fig 4:**
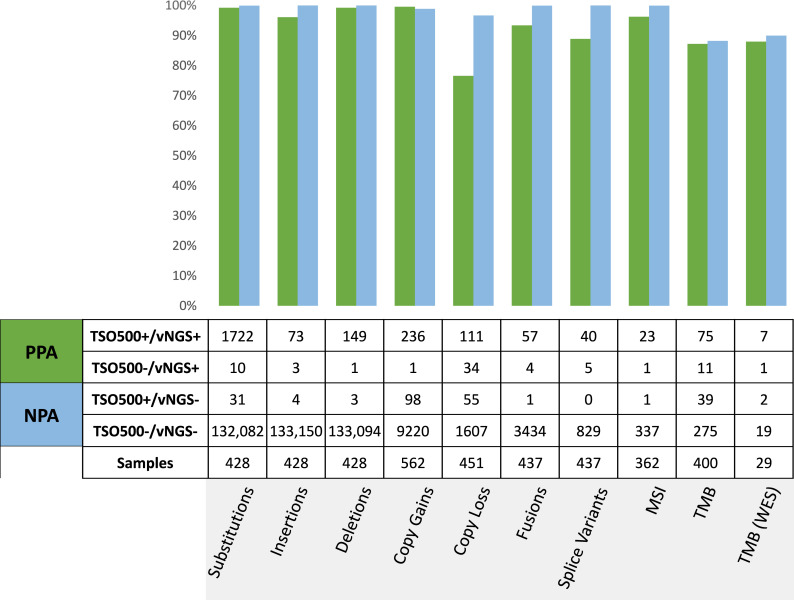
Concordance summary across all variant classes as positive percent agreement (PPA, green bar) and negative percent agreement (NPA, blue bar). The analytical accuracy of TSO500 was evaluated using clinical FFPE samples obtained from patients with a variety of tumor types to assess agreement with an orthogonal validated NGS assay (vNGS). Data was aggregated at the variant level for substitutions, insertions and deletions, at the gene level for copy gain, copy loss, fusions and splice variants, and case level for MSI and TMB.

The small variant comparison study evaluated a test set of 428 qualified specimens. A total of 1151 unique somatic small variant positions from 88 genes, including 83 unique insertions and 139 unique deletions from 32 genes were included in the study. Positive percent agreement (PPA) and negative percent agreement (NPA) was calculated for all small variant alterations (Fig 4, S11 Table in S1 File) between the TSO500 and the NY CLEP-approved OmniSeq Comprehensive Assay (OCP) using AmpliSeq chemistry, the Oncomine Comprehensive panel and Ion Torrent S5XL sequencing [3, 4].

In total, 125 genes and >133K bp of overlap sequences was assessed in the accuracy study providing platform-level performance for detection of somatic variants ranging from 2–100% VAF. All positions harboring Tier I and II SNV alterations are included in the overlap regions. Differences in indel sensitivity of the OCP vNGS (10% VAF cutoff) and indel variant sequence positions (+/- 1bp) were accounted for in this data set. Differences not due to low VAF were limited to variants of unknown significance and were expected based on differences in TSO500 pipeline and vNGS methods. Accuracy studies for the 1151 known OCP substitution positions demonstrated 99.2% PPA (1722/1734) and 99.9% NPA (132,082/132,120). In total, 88 of the 125 genes had a substitution evaluated for accuracy between the two platforms, ranging from 1 variant (n = 14 genes) to 218 total variants in TP53. Specific review of the 249 Tier I variant positions resulted in 100% PPA for the six genes evaluated. Only one substitution position harboring a Tier I variant (chr3:178952084) was TSO500+/vNGS-. This variant, *PIK3CA* H1047Y, was detected in TSO500 at 5% VAF and was not called by the OCP assay due to low base quality. Although manual review of the vNGS BAM demonstrated that the variant was present, we did not reclassify this variant as a true positive. The overall NPA across all Tier I substitution positions evaluated is > 99.99% (2,247 of 2,248).

Non-Tier 1 TSO500+/vNGS- substitutions occurred at <0.024% frequency (31/133,854 total calls). These discordant substitutions were investigated and categorized into three classes: 1) Assay issues, 2) Evidence of mutation in BAM, and 3) No evidence of mutation in BAM. Each variant was manually reviewed at the sample sequencing QC level, variant QC level, BAM view level, and genomic context (i.e., repetitive regions) for class assignment to explain discordance (S12 Table in S1 File). The 31 non-Tier I TSO500+/vNGS- discordant substitutions (false positives) represented unique variant positions across 19 genes. No genomic position had greater than one discordant call. 58% (18/31) had VAFs detected by TSO500 at <7% and were believed to be discordant because they have allele frequencies approaching the OCP 5% VAF threshold for substitution detection. The remaining 13 TSO500+/vNGS- variants ranging from 17–99% VAF (23–1778 ALT reads) averaged 479x read depth and passed all quality thresholds for variant calling. The TSO500+ *ATM* Q368* substitution mapped to an OCP blacklist position (amplicon end) and therefore could not be called by the OCP pipeline. Eight of the TSO500+/vNGS- substitutions were visible in the OCP BAM files, but were not called by the OCP pipeline. These include one *BRCA2* variant in a polyA tract and three (*PTCH1*, *NF1* and *RB1*) in regions with <20x coverage. Four substitutions (*TSC2*, *BRCA2*, *RB1* and *KIT*) were clearly visible in the OCP BAM but not called. Collectively these nine substitutions show evidence of the mutation in the OCP BAM and are believed to be TSO500+/vNGS+. The 4 remaining TSO500+/vNGS- substitutions demonstrated no evidence of the variant in the OCP BAM, though *WT1* S138C had <20x coverage and a *NOTCH1* D660N variant was near a large indel that was called TSO500+/vNGS+. Sanger sequencing of these four variants resulted in both *NFE2L2* substitutions being detected, but the *NOTCH1* and *WT1* variants not detected, resulting in 50% of these TSO500+/vNGS- substitutions being considered true positives.

The 10 non-Tier I TSO500-/vNGS+ discordant substitutions (false negatives) were unique variant positions across 9 genes (S12 Table in S1 File). Five of the 10 variants considered wild type by TSO500 demonstrated evidence of the mutation, but did not pass TSO500 variant calling quality metrics due to either low variant quality or low coverage depth. Two OCP substitutions (*CDKN2A* W15X and *TP53* E258Q) in complex regions with adjacent deletions (called by TSO500 and OCP) were not evident in TSO500. The complexity of these two regions is believed to have resulted in the discordant results in these cases. The three remaining TSO500-/vNGS+ substitutions with no evidence of a TSO500 mutation were OCP positive variants called at 10 and 11% VAF, including an *ATM* F357L variant located in a homopolymer T region. The TSO500-/vNGS+ *GATA2* H323Y substitution did not have visible evidence of the variant in the OCP BAM and is likely a false positive OCP call.

Accuracy studies for known indels (≤25 bp) demonstrated 96% PPA for insertions (73/76) and 99% for deletions (149/150), with both small variant types exceeding 99.9% NPA across the 125 genes evaluated. Included in this cohort were 6 samples with known Tier 1 MNVs (*BRAF* V600K and *KRAS* Q61K) that were detected 100% with no false positives. There was one discordant TSO500-/vNGS+ deletion (*RB1* T5PfsX60) that is in a homopolymer track and potentially a vNGS false positive. There were a total of three discordant TSO500-/vNGS+ insertions. The *FBXW7* 338_339delinsGT (V113G) was not detected by TSO500 across 12 replicates, but is located in a (AGG)7 repeat region and is believed to a true negative. Similarly, the *BRCA2* 6556_6557insA (S2186YfsX3) was called TSO500 wild type across ten replicates and the *CDH1* 44_46dup (L15dup) was called TSO500 wild type across three replicates. Both insertions are believed to be true negatives.

The majority of discordant indels calls were TSO500+/vNGS-. Given the reduced indel sensitivity of the Ion Torrent technology, it was anticipated that the Illumina sequencing chemistry would detect additional indels. The 7 discordant indels were unique, with three TSO500+/vNGS- deletions ranging from 15–27% VAF, and four TSO500+/vNGS- insertions ranging from 18–71% VAF. Six of the TSO500+/vNGS- indels were visible in the OCP BAM files but were not called by the OCP pipeline. *DNMT3A* R882_L883insC was near an OCP amplicon end and therefore not called by OCP pipeline. *BRCA2* N1784fs resides in a polyA tract and was not called by OCP pipeline. *ERBB2* A775_G776insL was detected by OCP but annotated differently leading to the discordant result. Three indels (GATA3 G144fs, GATA3 F410fs, and PTEN E43_Y46delinsD) were clearly visible in the OCP BAM and their vNGS- status could not be explained by their genomic context or position within the OCP target amplicons. Collectively these six indels (Complex—deletion inframe (n = 1), Deletion–Frameshift (n = 1), Insertion–Frameshift (n = 2), and Insertion—In frame (n = 2)) show evidence of the mutation in the OCP BAM and are believed to be true positives. The remaining TSO500+/vNGS- indel (*GATA3* C318fs) demonstrated no evidence of the variant in the OCP BAM. This large deletion (c.954_991del) includes 38 bp of sequence and could not be confirmed by orthogonal testing due to lack of remnant material. As a summary of small variant accuracy, the overall >99% PPA and >99% NPA with the vNGS assay demonstrates performance acceptable for clinical use and met the acceptance criteria of >95% PPA for substitutions, 90% PPA for insertions and deletions, and 99% NPA for all small variants.

### Copy number alterations (CNA)

From a clinical perspective, CNA evaluation reports amplification (copy gain) for oncogenes and deletion (copy loss) for tumor suppressor genes. The CNA comparison study evaluated a test set of 562 specimens for gain and 451 for loss between the TSO500 and the OCP vNGS assay. Though the TSO500 assay is designed to detect copy number fold change in 59 genes, the copy gain concordance studies were limited to 17 genes for which orthogonal data was available: *AR*, *CCND1*, *CCNE1*, *CDK4*, *CDK6*, *EGFR*, *ERBB2*, *FGFR1*, *FGFR2*, *KIT*, *KRAS*, *MET*, *MDM2*, *MYC*, *MYCN*, *PDGFRA* and *PIK3CA*. Copy loss studies were restricted to *ATM*, *BRCA1*, *BRCA2*, and *PTEN*. Both the concordance of TSO500 and vNGS FC values, and PPA/NPA agreement of copy number calls at the TSO500 cutoffs were calculated (Fig 4, S13 Table in S1 File). A total of 10,827 CNA calls, including >200 for gains and >100 for losses were included and demonstrated an overall 99.6% PPA (236/237) and 98.9% NPA (9,220/9,319) for copy gains, and 76.6% PPA (111/145) and 96.7% NPA (1,607/1,662) for copy loss. High correlation (R^2^ >0.91 and R>0.96) of copy gain fold change values (range 2.2–117) between the two platforms was observed for the 17 genes studied (S1 Fig in S1 File).

On an individual gene basis, copy gain PPA was 100% for all genes except *MYC* (94.4%). The lowest NPA was *CCND1* at 95.9%. Copy gain concordance was directly related to fold change. At TSO500 FC>5, 100% samples (178/178) at this value or higher were called copy gain by the comparator OCP vNGS. At TSO500 FC>4, 205/210 samples (97.6%) were called copy gain by the comparator vNGS. At TSO500 FC ≥3.2, 228/252 samples (90.5%) were called copy gain by the comparator vNGS. Between TSO500 FC 2.2 –<3.2, 236/336 samples (70.2%) were called copy gain by the comparator vNGS. To account for this lower sensitivity, a CNA in this range is denoted as copy “gain–indeterminate” and implies that the TSO500 assay data provides some but not unambiguous evidence that the gene fold change exceeds the threshold for identifying copy gain. At TSO500 FC <2.2, only 8/9227 samples (0.09%) were called copy gain by the OCP vNGS.

For Copy loss, the overall PPA for FC≤0.7 was 77% and overall NPA was 97%, ranging from a low of 50% PPA for *BRCA1* to 100% PPA for *PTEN*. The underlying *BRCA1* TSO500 fold change values demonstrate concordance to OCP values for 26 of 29 putative copy loss samples tested, with 15 FC values just above the 0.7 FC cutoff (median = 0.77, range 0.704–0.96). The lowest NPA was *PTEN* at 90%. The average estimated tumor purity for all copy loss samples was 73%, compared to 65% for no loss (n = 1662) and ranged from 30–100%. At FC≤0.5, which is cutoff for loss, 100% samples (29/29) were called copy loss by the comparator OCP vNGS. To account for lower sensitivity between FC >0.5–0.7, a CNA in this range is denoted as copy “loss–indeterminate” and indicates that the TSO500 assay data provides some but not unambiguous evidence of a deletion of the gene indicated. The results here and in the LoD study confirmed that copy loss in tumors with <50% estimated tumor purity are not reliably detected.

Investigation of the 22 TSO500+/vNGS- samples with TSO500 FC >3.2 revealed a case which was clearly an outlier. This case had a FGFR1 OCP CR of 0.12, which was significantly lower than the TSO500 FC of 3.73. Review of the OCP CN data for this case demonstrated large genomic instability, ranging from 0.01 to 323 CR, with multiple CN gains and losses. There is no information on tumor ploidy available to assess if this genomic characteristic may lead to the discordance. The remaining 21 cases had average TSO500 FC of 3.7 (range 3.23–4.49) and average OCP CR of 2.93 (range 2.1–3.63). We compared the TSO500 FC with the OCP FC to assess degree of correlation for these 21 cases (S14 Table in S1 File). The result was a Pearson correlation (R) = 0.57, with a high level of statistical significance (p value = 0.00743) demonstrating that although the final result (gain vs not gain) was discordant, the underlying quantitative values between the two assays were in close agreement for these samples. A review of the 21 samples showed that 70% were lung and breast CA, which is not surprising as these are the two most studied disease in the validation cohort (46%). A review of the genes show that MYC, CCND1 and MDM2 comprised of 64% of the TSO500+/vNGS- discordant CN gain cases. There were no samples with Tier 1 genes (*MET* or *ERBB2*) in this discordant cohort.

### Microsatellite instability (MSI)

The qualitative output for MSI (MSI-High vs. MS-Stable) in the TSO500 (130 potential repeat regions) and vNGS assay were evaluated for concordance. The orthogonal NY CLEP-approved vNGS assay uses custom amplicon libraries and Illumina sequencing to targeting 29 homopolymer tandem repeat regions [32]. PPA and NPA of MSI status between the two assays was calculated for 362 qualified samples studied (Fig 4, S15 Table in S1 File), including a subset of CRC (10%) and endometrial cancer (4%) specimens.

PPA for MSI-High was 96% and NPA was >99%, meeting the target for acceptable sensitivity (85% PPA) and specificity (95% NPA). In total, 10 tumor types were detected by both assays as MSI-High: Esophageal Cancer, Colorectal Cancer, Prostate Cancer, Small Intestine Cancer, Thymic Cancer, Stomach Cancer, Breast Cancer, Squamous Cell Carcinoma of the Skin, Uterine Cancer, and Unknown Primary Cancer, with all MSI-High samples also presenting as TMB-High. 11 of the MSI-High were CRC, with an overall 31% positive rate, with 50% of stomach cancers (3 of 6) also MSI-High. All lung cases (n = 111) were MS-Stable, consistent with prevalence rates of MSI across tumor types [50]. The TSO500-/vNGS+ discordant case was a CRC sample with 18% MSI unstable sites, which is just below the 20% MSI cutoff. Similarly, the TSO500+/vNGS- discordant case was a breast sample with 22% MSI unstable sites, which is just above the 20% MSI cutoff. This case was called MSI inconclusive by the vNGS comparator. Since the TSO500 assay targets different homopolymer regions than MSI-NGS vNGS comparator, it is plausible to expect outlier results in a few cases. In summary, the high overall PPA (96%) and NPA (99%) across the 368 clinical samples evaluated supports accurate TSO500 MSI detection at the 20% unstable sites cutoff.

### Tumor mutational burden (TMB)

The analysis for concordance of TMB-High detection by TSO500 (1.33 Mb region covered) at the 10 mut/Mb cut point was performed using a NY CLEP-approved vNGS targeted panel (1.17 Mb region covered) [47] and an NCI-validated whole exome sequencing (vWES) assay as comparators [51]. 429 qualified samples representing 34 tumor types were evaluated, with 29 for WES. TMB concordance results at the 10 mutations per megabase cut point between TSO500 and the comparator assays are summarized (Fig 4, S15 Table in S1 File). The PPA ranged from 87–88% and the NPA ranged from 88–90%, demonstrating consistent performance between the comparator platforms and meeting the target 85% PPA and NPA criteria. Four of the 11 TSO500-/vNGS+ discordant cases had TMB values of 9, just 1 mutation below the cut point value. Similarly, the one discordant TSO500-/vWES+ case had a TSO500 TMB value of 8 and the WES TMB value was 13. 11 of the 39 TSO500+/vNGS- discordant calls (28%) had vNGS TMB results between 8 and 9.9, again just below the 10 mut/Mb cut point.

**Fig 5:**
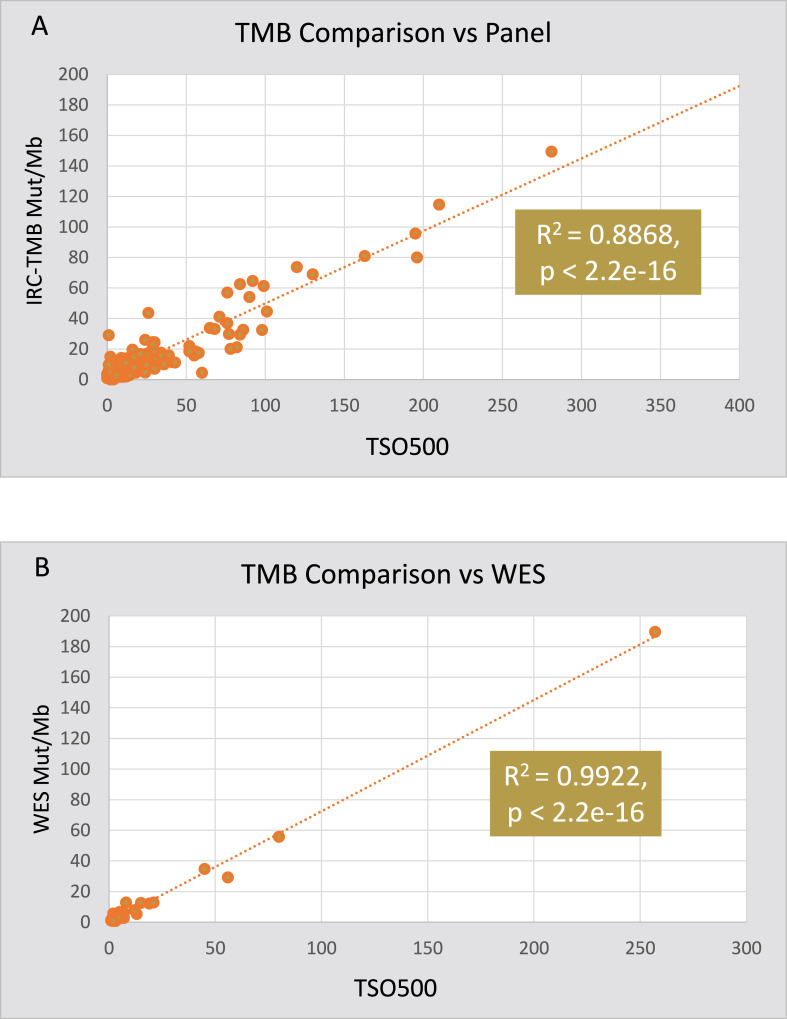
Scatter Plot and correlation of TSO500 TMB score against (A) vNGS panel and (B) vWES.

To assess the underlying quantitative TMB values for agreement and to further support analytical concordance, coefficient of determination (R^2^) of TMB values (0–281 mutations/Mb) for 400 samples generated by TSO500 and the vNGS were evaluated (Fig 5A). In addition, a Pearson correlation coefficient (R) was applied. Both concordance measurements (R^2^ = 0.8868 and R>0.94) demonstrates a strong TMB quantitative relationship between TSO500 and the vNGS. Similar analysis of TMB concordance between TSO500 and the vWES assay showed a high Pearson correlation (R = 0.99) and coefficient of determination (R^2^ = 0.9922) for the 29 samples included in the study (Fig 5B). Of the 112 samples (28%) called TMB-High, 38% were NSCLC, 14% CRC, 12% melanoma and 9% breast cancer (S16 Table in S1 File). This rate is higher than the expected prevalence in the intended use population but samples were intentionally selected to over-represent TMB-High cases for the purposes of the study. For tumor types with > 4 TMB-High samples, overall incidence of TMB-High was highest for HNSCC (75%), melanoma (68%), stomach and thymic cancer (50%), CRC (41%), NSCLC (36%) and bladder (33%). Tumors with low or no TMB-High called include ovarian (n = 13, 0%), brain (n = 9, 11%) and mesothelioma (n = 8, 0%).

### Fusions and splice variants

Overall there were ten clinically relevant overlapping genes between TSO500 and the OmniSeq Comprehensive vNGS assay for the detection of fusions and splice variants from RNA, which included 437 qualified samples from 35 different tumors and reference cell lines. Concordance results between TSO500 and the comparator assay for fusion and splice variant calling are summarized (Fig 4, S17 Table in S1 File).

Briefly, the fusion results demonstrated an overall 93% PPA (57/61) and >99% NPA (3434/3435) for a total of 3,496 observations across the eight genes studies. These values met the target for acceptable sensitivity (85% PPA) and specificity (99% NPA). The samples were primarily lung cancer (67%) with 10 other tumor types representing the remaining cases. Tumor purity ranged from 20–100% with necrosis ranging from 0–20%. There were a total of 4 samples which were TSO500-/vNGS+; two for *ALK*, one for *FGFR3* and one for *RET*. The vNGS amplicon-based read counts for the missed ALK fusions (187 and 2127) were much lower than the average positive ALK fusion positive read count of 76,064, suggesting that the hybridization capture based TSO500 may have lower sensitivity than the amplicon based vNGS assay. Similarly for *FGFR3*, the vNGS amplicon read count of 8,027 for the TSO500 false negative sample was much lower than the average *FGFR3* read count of 245,412. No remnant sample was available for orthogonal testing of the suspected single TSO500 false positive *RET* (*KIF5B-RET*) call which does involve a fusion partner specifically targeted by the vNGS assay (*KIF5B-RET*.K15R12). This sample had 5 unique TSO500 alternate reads which is the threshold for a fusion call. What may be surprising is the lack of novel fusions called by TSO500 as represented by this one TSO500+/vNGS- result. Given that the TSO500 hybridization capture approach does not require knowledge of the fusion partner, there was an expectation of additional TSO500+/vNGS- cases detected across the >400 tumor cases studied.

The splice variant results demonstrated high TSO500 concordance to vNGS with an overall 89% PPA and 100% NPA for a total of 874 observations for *MET* Exon 14 and *EGFR* Exons 2–7 Skipping. There were a total of 5 TSO500-/vNGS+ discordant calls (1.5%) representing 3 *MET* samples and 2 samples for *EGFR*. Each sample did have TSO500 splice variant reads detected but were below the 8 unique read threshold required for reporting. The average vNGS amplicon-based reads for the TSO500 negative samples was 11,562 for *EGFR* and 1,160 for *MET*. The *EGFR* value is 5.3% of the average positive *EGFR* read count (216,276) and 1.3% for *MET* (89,092), indicating that these TSO500-/vNGS+ calls are below the lowest limit of TSO500 detection. A review of the histologies and tissue QC specifications did not indicate a bias to tumor type, necrosis, tumor purity or cellularity.

The Seraseq® FFPE Tumor Fusion RNA v4 Reference Material is engineered with 16 fusions and 2 splice variants targeting 18 different gene partners. Of the 55 genes evaluated for fusion and the 2 targeted for splice variants by TSO500, all are represented in the reference standard at fusion copies ranging from 104–414 transcripts per ng RNA (based on ddPCR). TSO500 evaluation of Seraseq® FFPE Tumor Fusion RNA v4 Reference Material resulted in >97% accuracy for detection of the expected structural variants across 12 replicates with 60 ng RNA input (S18 Table in S1 File).

### Precision

Repeatability and reproducibility of alterations associated with Tier I mutations and platform-wide alterations, including agreement for small variants, CNA, MSI, TMB, fusions and splice variants were evaluated in multiple sequencing runs ranging from 36 to 72 samples per batch. Repeatability (5 replicates intra-run under same conditions) and reproducibility of inter-run replicates (3–49 replicates under different conditions) were assessed and compared across two different sequencers, five different reagent lots (combinations of assay and sequencing reagents), across multiple days (n = 13) by multiple operators (n = 3) with rotating barcodes (S19 Table in S1 File). 134 specimens representing 128 tumors and 6 QC controls were evaluated for precision separately for DNA (n = 90) and RNA (n = 44). Precisions studies assessed assay variability from nucleic acid library preparation through analytical pipeline analysis.

A total of 59 samples with sufficient nucleic acid yield had Tier I alterations. Within the assessment of repeatability and reproducibility for Tier I mutations, all replicates were called for a 100% positive call rate across all conditions except for *ALK* fusions and *MET* exon 14 skipping (S20 Table in S1 File). The *ALK* fusion precision study included three samples at the fusion positive cut point of 5 alternate reads which led to a lower overall positive call rate of 87%. The single *MET* exon 14 discordant sample failed RNA-seq QC.

**Fig 6:**
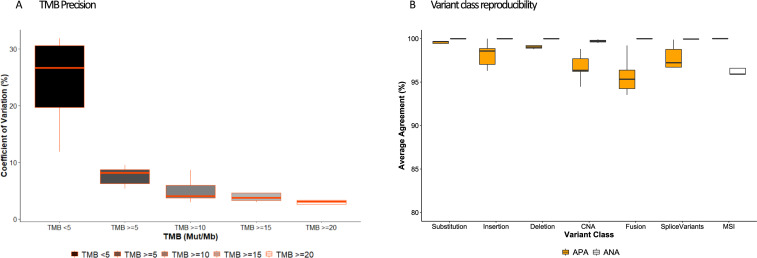
Precision summary across variable conditions including within (intra-) and between (inter-) runs, operators, days, instruments, reagent lots and barcodes. A) TMB precision was assessed using %CV of the TMB score across replicates for samples bordering consensus the TMB-High (>10 Muts/Mb) cut off. B) Imprecision in variant detection assessed using average positive agreement (APA, orange) and average negative agreement (ANA, white) within replicates for all variant classes.

TMB as a Tier I immune oncology biomarker with a quantitative result was evaluated for precision by assessing the variability of TMB values (mutation/Mb) detected at 5 mut/Mb intervals near the 10 mut/Mb cut point for TMB-High (S21 Table in S1 File). The overall CV at a TMB of ≥5 was below the acceptance criteria of 12.5% demonstrating precision in TMB values across the 89 unique samples studied (Fig 6A). Additionally, as the TMB magnitude increased, the overall CV trended downward, demonstrating increasing precision with increasing TMB values.

To study platform-wide precision across all variant classes and alterations, replicate studies were designed to assess positive and negative call rates with samples tested under various conditions including within (intra-) and between (inter-) runs, operators, days, instruments, reagent lots and barcodes. The samples began with extracted nucleic acids and spanned variants of all ranges. As with the Tier I variant positions, the platform-level repeatability and reproducibility showed high overall average positive agreement (APA) and average negative agreement (ANA) across variant classes (S22 Table in S1 File, Fig 6B) calculated by averaging the positive call rates and negative call rates from all the replicate measures across the variable conditions. The platform-level study included a total of 3699 unique substitutions, 453 indels, 37 CNA genes, and 29 structural rearrangements in the variant set across the samples. Small variants, including substitutions, insertions and deletions were further evaluated for precision at VAFs ranging from ≥ 0 to ≥ 15. The positive call rates for each small variant type were >99.4% at the panel-wide 5% VAF threshold for detection (S23 Table in S1 File).

Precision was also evaluated at the sample level for the 17 copy gain and 4 copy loss genes evaluated for accuracy. The results demonstrate 100% positive and negative call rates across 124 of 125 samples evaluated for gain at the >2.2 FC cut point (S24 Table in S1 File) and for 14 of 14 (100%) samples for loss (S25 Table in S1 File) at the <0.7 FC cut point. Reproducible measurement of the underlying quantitative FC values is also supported by the minimal standard deviations and CV of the mean values.

MSI precision demonstrated 100% positive and negative call rates across the 5 samples evaluated for MSI-H and 6 samples for MS-Stable (S26 Table in S1 File) at the >20% unstable site cut point. Reproducible measurement of the underlying quantitative unstable site values is also supported by the minimal standard deviations and CV of the mean values.

### Analytical sensitivity (LoD) assessment

The Limit of Detection (LoD) of alterations was evaluated at the platform level and at select variant positions. The LoDs for small variants is based on VAF and for non-small variant alterations based on tumor purity. All LoD studies were performed at the lower recommended DNA (40ng) and RNA (20ng) input using diluted and undiluted clinical FFPE tumor (n = 78) and contrived samples (n = 1) selected for each of the variant classes. For each sample, three—six levels of dilution (neat to 1:32) dependent on starting tumor purity, alteration frequency and sample availability were evaluated with and without replication. Dilutions were performed by mixing normal DNA or RNA from FFPE specimens with the clinical FFPE specimens. Select FFPE specimens were also used to evaluate the LoD of specific mutations with Tier I evidence of clinical significance. The established analytical sensitivity ranges were confirmed at ≥95% call hit rate with FFPE clinical cases on a per variant level for Tier I alterations, and using all somatic variants identified in FFPE clinical cases representing a range of VAFs for hotspot and non-hotspot positions for platform-wide assessment (Table 1).

**Table 1:** Analytical sensitivity (LoD VAF) for small variants (SNVs and indels).

Variant Class/Mutation	Mean LoD VAF (100% Hit Rate)	Range of Minimum VAF	Samples	Total alterations	Unique alterations
Overall	5.1%	2.7% - 12.1%	36	4685	2775
Substitution	5.2%	2.7% - 12.5%	36	4266	2441
Deletion	3.4%	1.6% - 5.7%	36	335	259
Insertion	4.2%	2.0% - 15.8%	36	84	75
*EGFR* Exon 19 E746_A750del	3.6%	1.0–8.5%	2	2	2
*EGFR* T790M	1.3%	0.86–1.8%	2	2	1
*EGFR* L858R	1.5%	1.50%	1	1	1
*KRAS* G12A	2.3%	2.30%	1	1	1
*BRAF* V600E/K	2.4%	1.7–3.8%	2	2	2
*FGFR3* S249C	2.3%	2.30%	1	1	1
*PIK3CA* E545K/Q546R	2.1%	1.9–2.4%	3	3	2
*PDGFRA* D842V	1.4%	1.40%	1	1	1

Analytical sensitivity of MSI-H, TMB-H, copy gain, copy loss, fusions and splice variants was also evaluated by testing clinical FFPE cases of several tumor types diluted with normal FFPE DNA to achieve targeted detection levels of alterations at low tumor purity. The LoD is based on the average minimum tumor purity required for a positive call across all samples studied (Table 2).

**Table 2:** Analytical sensitivity (LoD tumor purity) for copy gain, copy loss, MSI, TMB, fusions and splice variants.

Variant class/Mutation	Mean LoD Tumor Purity (100% Hit Rate)	Range of Minimum Tumor Purity	Samples	Total alterations	Unique alterations
Copy gain (FC >5)	16%	2–40%	12	26	20
Copy Gain (FC 2.2–5)	28%	21–33%	3	3	2
Copy Loss (FC <0.7)	73%	45–90%	3	5	3
MSI-H	19%	10–34%	5	5	1
TMB-H (> = 10 mut/Mb)	14%	5–48%	12	12	1
Fusions	6%	1.8–14%	6	6	6
Splice Variants	5.60%	3.75–7.5%	2	2	2
*RET* Fusion	6.25%	6.25%	1	1	1
*NTRK3* Fusion	5%	5%	1	1	1
*ALK* Fusion	14%	14%	1	1	1
*ERBB2* Copy Gain	15%	15%	1	1	1

## Discussion

Comprehensive genomic profiling using targeted NGS is a powerful tool for the detection of genomic alterations and other tumor biomarkers that support precision medicine. Unlike whole exome or whole genome sequencing, targeted CGP focuses on genomic regions known to have strong biologic or clinical relevance, thereby generating greater sequencing depth to confidently identify low frequency small variants and coverage uniformity to reliably evaluate copy number and tumor properties such as MSI and TMB. Targeted CGP also has the advantage that it can be employed without a matched non-tumor specimen, and is well-suited to assess low-quality clinical tumor samples with heterogenous characteristics (variable necrotic and neoplastic content) and minimal nucleic acid input. Testing with a NGS-based CGP assay also offers opportunities to replace single-gene tests performed either iteratively or in parallel, providing rapid comprehensive marker detection and better patient outcomes [52–55]. With the growing number of gene variants, signatures and pan-tumor biomarkers that are targetable with FDA-approved therapies (e.g., TMB, MSI, *ALK*, *BRAF*, *ROS1*, *NTRK*), targeted CGP is progressively considered as standard of care by many ordering oncologists and pathologists in the management of patients with advanced cancers. Clinical practice guidelines such as NCCN also recommends that biomarker testing on patients be performed “via a broad, panel-based approach” that can consolidate multiple molecular testing modalities into a single test to preserve frequently limited tumor tissue and obviating the need for repeat biopsies [56–59]. Furthermore, NCCN guidelines supports CGP as an efficient tool to improve patient access to clinical trials where CGP does not identify on-label therapy options [60].

Collectively, these features make CGP attractive for screening patients for detection of multiple DNA and RNA variant types. Yet, widespread implementation is hampered by logistics in FFPE specimen management, inconsistent sample volumes for cost-effective testing, the bioinformatics resources required to generate sophisticated therapy and trial matching reporting, regulatory requirements, and the expertise needed to develop, validate and operationalize CGP assays that meet clinically actionable turnaround times. Given these challenges and the expansion of biomarker driven precision therapies, reference laboratories dedicated to expanded molecular testing using CGP are instrumental in the systematic and timely delivery of personalized medicine options to patients who may benefit from targeted or IO therapies.

Towards this end, we developed and validated a high-throughput targeted NGS-based CGP assay which incorporates workflow automation and supporting software systems designed to process up to 70 clinical samples in a single run with a turnaround time of <7 days from specimen receipt to report. This scalable NGS assay, based on the TSO500 hybridization-based DNA and RNA capture assay, enables detection of all classes of current and emerging clinically relevant alterations, with sufficient genomic footprint to accurately determine small variants in the coding sequence of 523 genes, copy number of 59 tumor related genes, fusions (both known and novel partners) and splice variants in 55 select genes, TMB and MSI. With over 50 actionable markers having FDA label or NCCN guideline therapeutic evidence, the assay is designed to identify specific variants and genomic signatures to facilitate therapeutic selection in patients with advanced or metastatic solid tumors. Moreover, the assay supports comprehensive trial matching by targeting over 250 genes with clinical trial associations, supporting both biomarker-driven clinical studies and drug development.

Five informatics solutions manage the critical steps in the test procedure and insure that quality control measures are taken to control and monitor assay performance for consistent and reliable results. These five solutions include 1) electronic order entry, sample accessioning and reporting (CONNECT); 2) specimen, reagent, instrument and workflow management (Clarity LIMS); 3) TSO500 analytic pipeline; 4) quality review, variant review, pathologist sign-out and report generation, (GenomOncology Pathology Workbench), and; 5) evidence curation for therapeutic associations and clinical trials (OmniSeq Knowledgebase). The IT/informatics solutions were developed under ISO 13485:2016, IEC 62304 A:2016, and ISO 14971:2019, and communicate via web services insuring that the process offers the highest degree of quality control from order entry to final report. Together with an automated workflow, the integrated systems coordinate the processing of 140 RNA and DNA libraries in a single sequencing batch, scalable to 560 per week for each NovaSeq 6000 sequencing system. The analytic pipeline, variant annotation engine and Pathology Workbench combine to generate clinical reports available for signout within 8 hours of sequencing.

We validated the assay performance using more than 700 FFPE tumor specimens, including reference controls with known molecular alterations at low frequency, and assessed the devices ability to process and accurately detect multiple variant classes in batch sizes from 36 to 70 specimens per sequencing run. Each variant class was independently evaluated across a broad range of variant frequencies and reportable values using only 40ng DNA and 20ng RNA as input. Although not every gene or variant position could be evaluated due to rarity of clinical specimens with low prevalence alterations and the millions of potential altered sites, 96 cancer-related genes with orthogonal measurements were evaluated. The overall performance of the assay was high with all sequencing runs passing the quality control metrics and >95% of all tumor samples passing library preparation and sequencing QC. Using UMIs, the assay demonstrated high specificity enabling the detection of rare mutation events without increasing the levels of sequencing artifacts which potentially leads to false positive results at high sequencing depth. Our studies demonstrate reliable detection at 2% VAF for Tier I variant positions and 5% VAF for all other genomic locations with >99% overall small variant agreement between methodologies (Table 3). The assay demonstrated 99% copy number PPA to results from the vNGS assay at a ≥2.2 FC threshold for copy gain, and 77% overall PPA ≤0.7 FC threshold for copy loss. Although specificity remained high (97%) at estimated tumor purity >50%, the lower overall copy loss PPA is primarily attributed to *BRCA1* discordance between the two platforms at the 0.5 to 0.7 FC range. There was 100% agreement for all *BRCA1* samples with <0.5 FC, providing confidence in the assay’s ability to detect focal chromosomal loss in this clinically relevant gene. At ≥20% tumor purity, the TMB and MSI immunotherapy biomarkers that rely on the analysis of multiple loci also demonstrated high concordance. Likewise, the RNA-seq test mode used for the identification of both novel and known fusions in *ALK*, *RET*, *ROS1*, *NTRK1*, *NTRK3* and *FGFR3* and splice variants targeting *EGFR* and *MET* exon skipping were highly concordant to the orthogonal NGS assay utilized. Variant calls across all mutation classes are reproducible within runs, between runs and across operators, days, reagent lots, batch sizes, barcodes and NovaSeq 6000 instruments.

**Table 3:** Concordance summary for all variant classes and biomarkers.

NGS	Passing Criteria	Genes/Loci	Marker	Samples	PPA	NPA
DNA-Seq	Tier I hotspots: ≥ 2% VAF	523	Substitutions	428	99%	>99%
	Non-hotspots: ≥ 5% VAF		Insertions		96%	>99%
			Deletions		99%	>99%
	≥ 2.2x fold change; 30% tumor purity	59	Copy gain	562	99%	99%
	≤ 0.7x fold change; 50% tumor purity	4	Copy loss	451	77%	97%
	≥ 20% tumor purity	521	TMB ≥ 10 mut/Mb	429	87%	88%
		130	MSI	362	96%	>99%
RNA-Seq		55	Fusions	437	93%	>99%
		2	Splice variants		89%	>99%

Although robust, the assay has limitations which were identified during the analytical study. As a qualitative assay without a recognized reference method or ‘gold standard’, accuracy or ‘correctness’ was estimated by comparison to predicate vNGS methods as measures of agreement (PPA and NPA). Therefore, this approach cannot directly calculate accuracy (sensitivity and specificity) or positive predictive value or negative predictive value, and is not a true measure of ‘correctness’ as both tests could agree and both be incorrect. To mitigate this potential issue, we report both positive and negative percent agreement to characterize test performance and utilized only validated orthogonal NGS assays as predicates. The hybridization-based approach requires a minimum of 20% tumor purity, 40ng DNA and 20ng RNA to provide enough unique molecules to reliably detect mutations at low frequency. Even though these inputs approach the lowest in the industry, the requirements may lead to insufficient material in a small percentage of specimens with low tumor mass, such as needle core biopsies. The assay validation for copy loss was limited to *BRCA1*, *BRCA2*, *ATM* and *PTEN*, with plans to validate additional genes in the future as reference materials and orthogonal testing become available. Our insertion and deletion study was restricted to the detection of indels to alterations ≤25bp which precludes reporting of rare but potentially deleterious or suspected deleterious indels >25bp. Further studies and pipeline enhancements are needed to address larger insertions and deletion up to 100bp. The studies revealed that MSI was the variant class most affected by low quality samples as demonstrated by higher within-sample QC failure rates compared to the other alteration types. The reason for failure is likely variability in library insert size with more degraded DNA associated with higher MSI invalid calls. The library preparation workflow is complex and labor intensive, requiring a 2-day process. Without automation, there is significant hands on-time from sample-to-sequencing which may hinder adoption due to the costs associated with liquid handling instrumentation. Although the analytical sensitivity of the TSO500 may be less than amplicon-based assays for fusion detection, the clinical performance and overall robustness of the test, and the potential to detect novel fusion partners, makes it a superior alternative to amplicon-based tests for most purposes.

In summary, the results of this extensive analytical study demonstrate that the comprehensive genomic profiling assay provides robust, accurate and precise detection of all major variant classes in addition to MSI and TMB signatures in solid tumors. Together with automation, supportive software systems, an optimized bioinformatics pipeline and high-throughput NovaSeq 6000 sequencers, the device can simultaneously process 70 samples and generate a comprehensive final report on each patient in less than a week from sample receipt. By incorporating both DNA and RNA sequencing from a single tissue sample, this well-validated comprehensive assay has the genomic coverage necessary for accurate selection of FDA-approved targeted and IO therapies, while also evaluating a large number of genes relevant to early phase drug development efforts that may have future clinical application.

## Supporting information

S1 File Supporting information. (XLSX)
